# Stronger stimulus triggers synaptic transmission faster through earlier started action potential

**DOI:** 10.1186/s12964-024-01483-3

**Published:** 2024-01-12

**Authors:** Zhuoyu Zhang, Rong Huang

**Affiliations:** 1 Neurological Department of Tongji Hospital, School of Medicine, Tongji University, Shanghai, 200333 China; 2 Neuroscience Research Center, Institute of Mitochondrial Biology and Medicine, Key Laboratory of Biomedical Information Engineering of Ministry of Education, School of Life Science and Technology, Xi’an Jiaotong University, Xi’an, 710000 China

**Keywords:** Synaptic transmission, Action potential, Intensity of external stimulus, Occurrence time of secretion, Amplitude of secretion

## Abstract

Synaptic transmission plays an important and time-sensitive role in the nervous system. Although the amplitude of neurotransmission is positively related to the intensity of external stimulus, whether stronger stimulus could trigger synaptic transmission faster remains unsolved. Our present work in the primary sensory system shows that besides the known effect of larger amplitude, stronger stimulus triggers the synaptic transmission faster, which is regulated by the earlier started action potential (AP), independent of the AP’s amplitude. More importantly, this model is further extended from the sensory system to the hippocampus, implying broad applicability in the nervous system. Together, we found that stronger stimulus induces AP faster, which suggests to trigger the neurotransmission faster, implying that the occurrence time of neurotransmission, as well as the amplitude, plays an important role in the timely and effective response of nervous system.

## Background

Fight or flight is the natural response when faced with danger [1, 2], which time window is very short to make effective decision timely. The underlying control system is the nervous system, which receives the external stimuli and processes these signals through action potentials (APs) and synaptic transmission within milliseconds. Briefly, external stimuli induce the APs to activate the voltage gated calcium channels [3, 4] for Ca^2+^ influx, which indirectly triggers presynaptic vesicles fusion with the plasma membrane [5–7] to release the neurotransmitters [8–10]. Apart from the above Ca^2+^ dependent secretion, in some types of neurons like the sensory neurons and sympathetic neurons, the APs can directly trigger vesicular secretion, independent of Ca^2+^ [11–13]. In other words, the APs are the upstream trigger of neurotransmission. After vesicular exocytosis, the released neurotransmitters then diffuse and bind with the postsynaptic receptors, inducing the postsynaptic current (PSC), either in excitatory or inhibitory form. This precise and complex network communication [6] between neurons is the basis to maintain the survival and development of animals with nervous system. Previous studies showed that stronger stimuli trigger larger neurotransmission signals [12, 14]. However, little attention is paid to whether the stronger stimulus would result to the faster occurrence of neurotransmission.

Combining the local electrical-field stimulus and postsynaptic patch-clamp recording, we capably triggered the presynaptic AP to evoke vesicle release and recorded the postsynaptic PSC. Consistent with the previous reports, stronger stimulus triggers larger amplitude of the PSC signal. What is more interesting is that stronger stimulus induces the presynaptic AP and postsynaptic PSC faster, suggesting that the faster PSC signal was regulated by the earlier started AP. Further, this phenomenon was repeated in hippocampal neurons as well, implying broad applicability that stronger stimulus triggers synaptic transmission faster through earlier started AP in the nervous system.

## Method

### Animals

The use and care of animals were approved and directed by the Institutional Animal Care and Use Committee of Xi’an Jiaotong University and Peking University and the Association for Assessment and Accreditation of Laboratory Animal Care. Rats were housed in an animal facility with a maximum of 5 rats/cage under a 12-h light/dark cycle at 22 ± 2 °C. Food and water were available *ad libitum*. For the animal use, the dorsal root ganglion (DRG) and spinal dorsal horn (DH) neurons co-culture needs one postnatal 3^rd^ day Sprague-Dawley rat (for DRG) and two 15^th^ day embryos of Sprague-Dawley rat (for DH) for each batch. The hippocampal neurons culture needs one postnatal 0^th^ day Sprague-Dawley rat for each batch. All recording needs at least three replicated batches.

### Cell culture of DRG, DH and hippocampus neurons

DRG neurons isolation and culture were performed as described [11, 12, 15, 16]. Briefly, the DRG ganglia were dissected and placed in cold L15 medium (Gibco). After removing the attached tissue, the ganglia were cut into 2–6 pieces and incubated in Dulbecco’s modified Eagle’s medium (DMEM)/F12 (Gibco) containing trypsin (0.3 mg/ml) and collagenase (1 mg/ml) for 40 min at 37 °C under 5% CO_2_. Then the pieces were washed twice with DMEM/F12 and dissociated into single cells by 8–10 bouts of trituration. The cell suspension was placed onto 0.1 mg/ml poly-L-lysine pretreated coverslips and maintained in Neurobasal supplemented with 2% B27 and 0.5 mM L-glutamine (all from Gibco).

Spinal DH neurons isolation and culture were performed as described [13, 15, 17]. Briefly, the spinal cord was dissected from 15^th^ day embryos of Sprague-Dawley rats and placed in cold D-hanks solution. After removing the attached tissue, the dorsal spinal cord was collected and cut into small pieces of about 1 mm^3^. After incubation in 0.25% trypsin solution for 15 min at 37 °C under 5% CO_2_, the dorsal horn pieces were washed twice with Neurobasal supplemented with 2% B27 and 0.5 mM L-glutamine (all from Gibco) and dissociated into single cells by 8–10 bouts of trituration. After that, the suspension containing DH neurons was placed onto the pre-cultured DRG neurons. All cells were maintained in the Neurobasal supplemented with 2% B27, 0.5 mM L-glutamine (all from Gibco), 10 ng/ml nerve growth factor (NGF), and 5 µM cytosine arabinoside for long term co-culture. The neurons were used for recording after 12 days co-culture.

Hippocampal neurons isolation and culture were performed as described [13, 18–20]. Briefly, the hippocampal brain tissue was dissected from neonatal 0^th^ day rats and placed in cold D-hanks solution. After removing the attached tissue, the hippocampal pieces were digested in 0.25% trypsin for 15 min at 37 °C under 5% CO_2_. Then the pieces were washed twice with Neurobasal supplemented with 2% B27 and 0.5 mM L-glutamine (all from Gibco) and dissociated by 8–10 bouts of trituration into single cells. Then the cells were placed onto 0.1 mg/ml poly-L-lysine coated coverslips and maintained in Neurobasal supplemented with 2% B27 and 0.5 mM L-glutamine (all from Gibco). Experiments were performed after 12 days culture.

### Electrophysiology

Postsynaptic currents (PSCs) were recorded under the whole-cell configuration using an EPC10/2 amplifier controlled by Patchmaster software (HEKA Elektronik) as previously described [12, 13]. The pipette resistance was controlled to ~ 10 MΩ and the membrane potential was clamped at − 70 mV for PSC recording. The standard external bath solution contained (in mM): 2.5 CaCl_2_, 1 MgCl_2_, 150 NaCl, 5 KCl, 10 H-HEPES, and 10 D-glucose, pH 7.4. The standard intracellular pipette solution for AP recording contained (in mM): 5 KCl, 4 MgCl_2_-6H_2_O, 145 k-gluconate, 5 EGTA, 10 H-HEPES, pH 7.2. Standard intracellular solution for PSC recording with CsCl containing (in mM) 4 Mg-ATP, 5 QX314, 153 CsCl, 1 MgCl_2_, 10 H-HEPES, pH 7.2. For PSC/AP recordings, local electrical-field stimulus impulse was applied via a laboratory-made bipolar microelectrode (diameter, 200 μm) connected to an electronic stimulator (Nihon Kohden, SEN-3201). The weak stimulus with “either small amplitude or short duration” must be just enough to trigger the PSC or AP response, which is a little bigger than the threshold for PSC or AP. The strong stimulus with “either large amplitude or long duration” is powerful to trigger the PSC or AP signal immediately after the artifact of electrical stimulus. However, it can’t be too strong to evoke multiple APs, which could bring new variables. All recordings were made at room temperature. Igor software (Wavemetrics, Lake Oswego, OR) was used for all offline data analysis. Series conductance and membrane conductance were used to monitor the seal condition during patch-clamp recordings.

### Statistical analysis

All experiments were performed with controls side-by-side and in random order and were replicated at least three times. Sample sizes are consistent with those reported in similar studies. Data are shown as mean + s.e.m. Statistical comparisons were made with the paired two-tailed Student’s t test as indicated. The distribution of the variables in each experimental group was approximately normal. All tests were conducted using Prism V8.0.1 (GraphPad Software, Inc.). Significant differences were accepted at *p* < 0.05.

## Results

### Stronger stimulus triggers the neurotransmission faster in sensory system

**Fig. 1:**
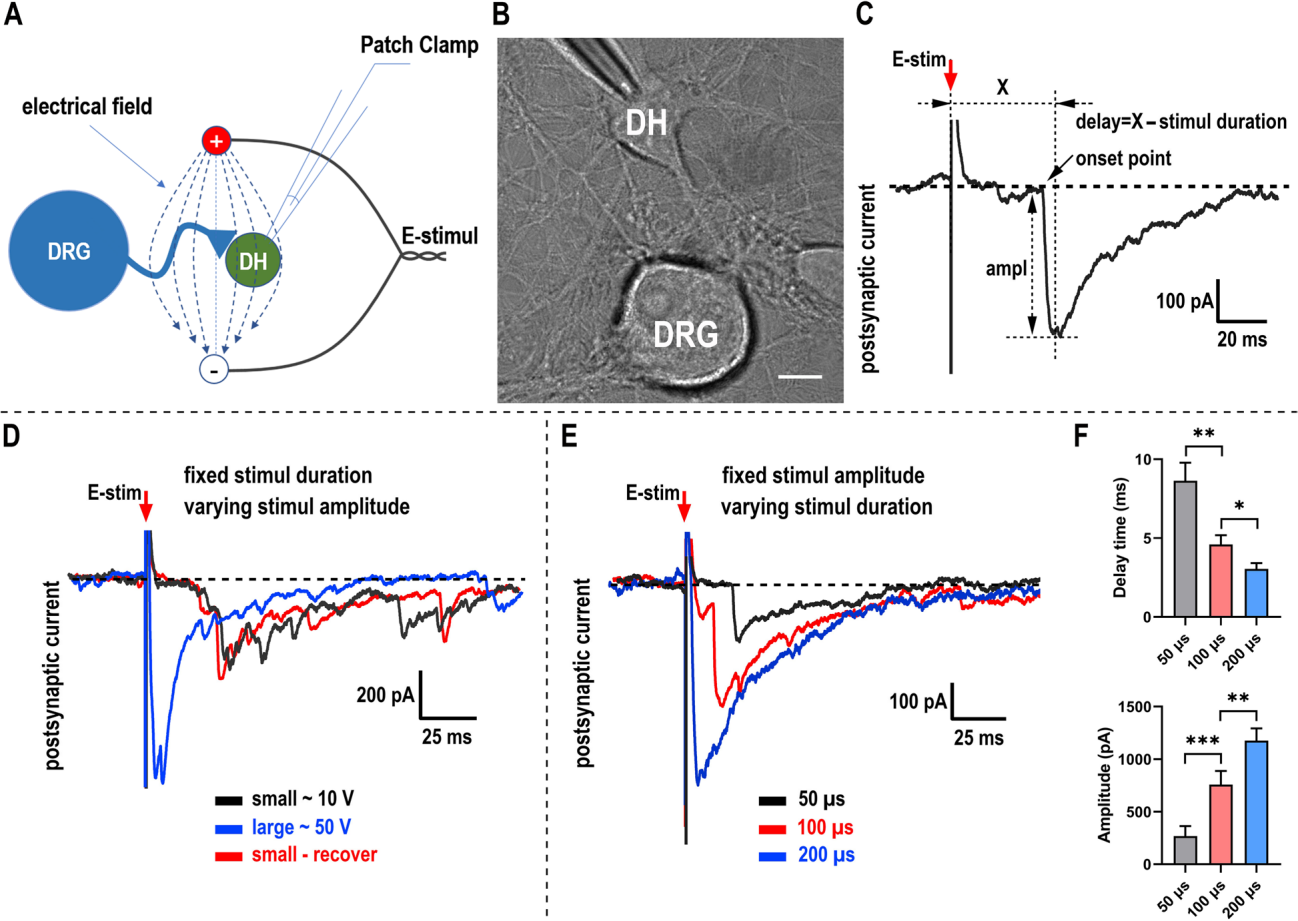
Stronger single pulse stimulus triggers the PSC signal faster in sensory system.** A **Model showing electrical field stimulus and patch clamp recording in sensory DRG-DH coculture system. **B** Image showing presynaptic DRG neurons and postsynaptic DH neuron with the patched glass electrode. Scale bar, 10 μm. **C** Definition showing the onset point and the amplitude of the PSC signal. To simplify the analysis, we analyzed and compared the delay time as the delta time from the onset of stimulus artifact to the peak of the PSC signal and then minus the duration of applied stimulus impulse. **D** Typical traces showing the onset point move according to the intensity of local stimulus [keep stimulus duration constant (100 μs), only change the amplitude (~10 V or 50 V)]. First weak stimulus (black), then strong stimulus (blue), last recover to weak stimulus (red). **E** Typical traces showing the onset point move according to the intensity of local stimulus [keep stimulus amplitude constant (~35 V), only change the duration]. First short-duration stimulus (50 μs, black), then intermediate-duration stimulus (100 μs, red), last long-duration stimulus (200 μs, blue). **F** Quantification of (**E**). 10 cells for 50 μs, 100 μs and 200 μs stimulation, respectively. All recordings in each panel were performed in the same one cell and aligned based on the onset of the stimulus. The time interval between different stimulus is at least one min. Data are shown as mean + s.e.m; paired Student’s t test for panel F; *p < 0.05; **p < 0.01; ***p < 0.001

To study the coupling between external stimulus and evoked neurotransmission signal, we cocultured the primary sensory dorsal root ganglion (DRG) neurons with spinal dorsal horn (DH) neurons and made patch-clamp recording on the postsynaptic DH neuron, using laboratory made bipolar stimulus electrode to apply local electrical-field stimulus (Fig. 1A and B). The stimulus intensity depends on the amplitude and duration of each applied square-wave impulse [13].

We first remained the duration of square-wave impulse unchanged and changed the amplitude solely. The large amplitude impulse (termed as strong stimulus) evoked larger postsynaptic current (PSC) than small amplitude impulse (weak stimulus) (Fig. 1D), which is consistent with previous reports [12]. Besides, it is interesting that the onset points (defined in Fig. 1C) of each PSC trace under different intensity of stimulus are not constant. The weak stimulus evoked PSC’s onset point is more away from the stimulus artifact than strong stimulus evoked PSC’s onset point (Fig. 1D). By switching the strong stimulus to weak stimulus, the onset point moves away from the stimulus artifact (Fig. 1D), which phenomenon is reversible in several repeats in the same one neuron. To make it more convincing, we kept the amplitude of square-wave impulse unchanged and changed the duration (50, 100, 200 µs) solely. Consistently, we got the same results that stronger stimulus (with longer duration) evoked PSC’s onset point is much closer to the stimulus artifact while weaker stimulus (with shorter duration) evoked PSC’s onset point is more away (Fig. 1E and F). For the PSC’s amplitude, it is positively related to the stimulus intensity. Overall, we found that stronger stimulus triggers larger synaptic transmission faster.

### Stronger stimulus triggers the AP more quickly in sensory system

**Fig. 2:**
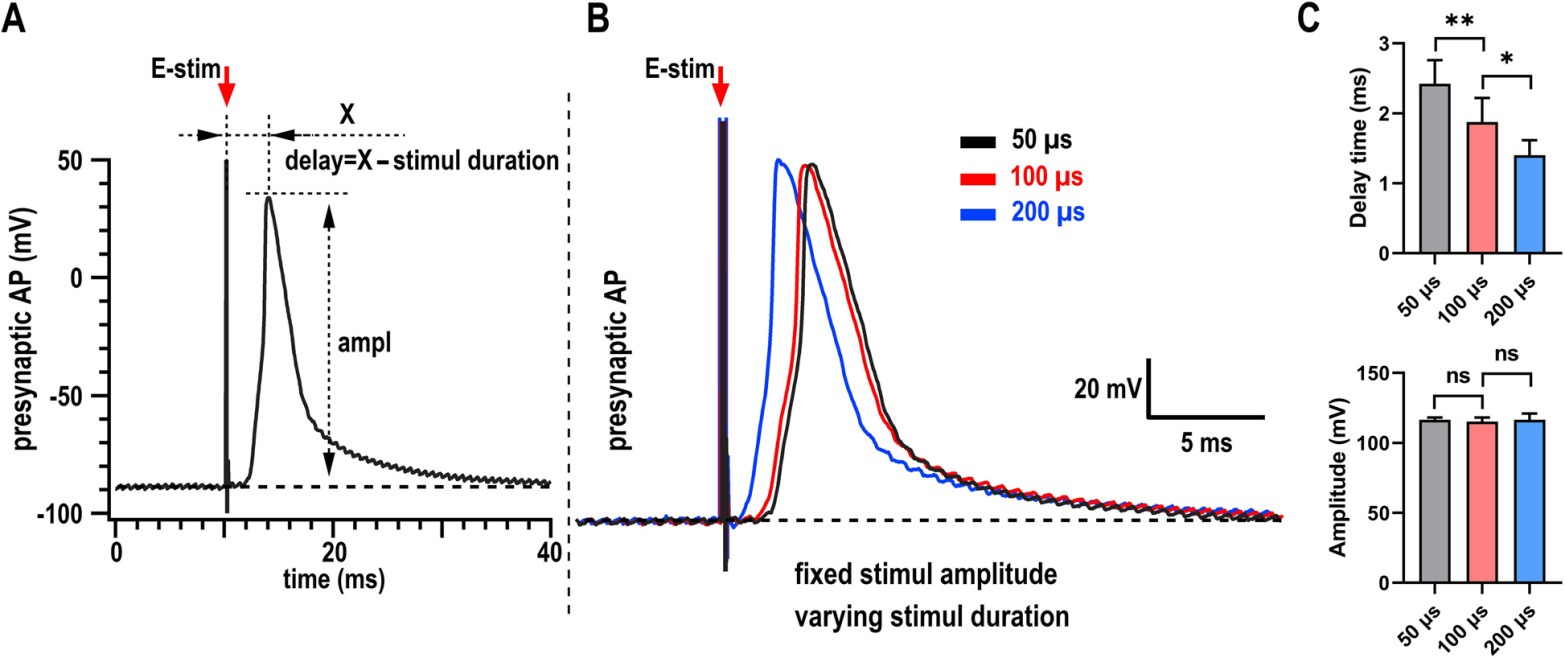
Stronger single pulse stimulus triggers the AP signal faster in sensory system. **A** Definition showing the delay time and the amplitude of the AP signal. We analyzed the delay time as the delta time from the onset of stimulus artifact to the peak of the AP and then minus the duration of applied stimulus pulse. **B** Typical traces showing the delay time move according to the intensity of local stimulus [keep stimulus amplitude constant (~35 V), change the duration]. First short-duration stimulus (50 μs, black), then intermediate-duration stimulus (100 μs, red), last long-duration stimulus (200 μs, blue). All recordings were performed in the same one cell and aligned based on the onset of the stimulus. The time interval between different stimulus is at least one min. **C** Quantification of (**B**). 9 cells for 50 μs, 100 μs and 200 μs, respectively. Data are shown as mean + s.e.m; paired Student’s t test for panel **C**; *p < 0.05; **p < 0.01; ns, not significant

The PSC signal results from presynaptic vesicles secretion, which is triggered by the presynaptic AP [7, 21]. Under stronger stimulus, we recorded earlier started PSC signal (Fig. 1D and E), which may be correlated to the secretion-trigger, i.e., the presynaptic AP. To reveal whether this earlier started PSC signal was regulated by the presynaptic AP, using the same stimulating and recording protocol, we kept the amplitude of square-wave impulse unchanged and changed the duration (50, 100, 200 µs) solely. Interestingly, the stronger stimulus (with longer duration) triggered AP more quickly (Fig. 2) in the same one neuron, which suggests to explain the underlying mechanism of the flexible onset points of PSC signals triggered by different intensity of stimulus. Nevertheless, there is no dramatical change for the amplitude of the APs evoked under different intensity of stimulus, indicating that the different amplitudes of neurotransmission were not triggered by the larger APs. Collectively, our data showed that stronger stimulus evoked AP more quickly than weaker stimulus, which suggests to further trigger the neurotransmission faster.

### Stronger stimulus triggers synaptic transmission faster through earlier started AP in hippocampal system

**Fig. 3:**
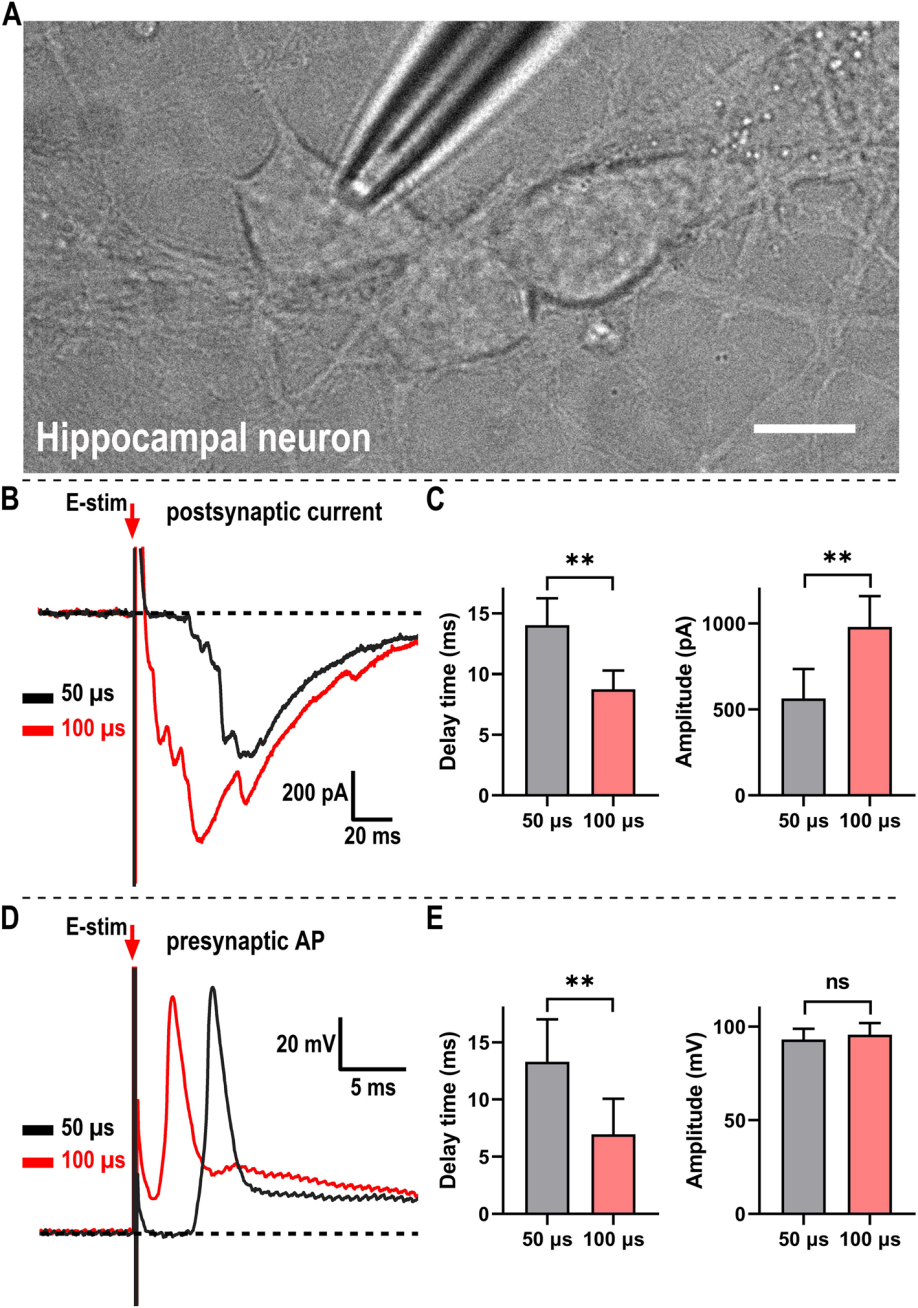
Stronger single pulse stimulus triggers the PSC and AP signal faster in hippocampal system.** A** Image showing patch clamp recording in cultured hippocampus neurons. Scale bar, 10 μm. **B** Typical traces showing the delay time of PSCs change according to the intensity of local stimulus [keep stimulus amplitude constant (~35 V), change the duration]. First short-duration stimulus (50 μs, black), then long-duration stimulus (100 μs, red). **C** Quantification of (**B**). 16 cells for 50 μs and 100 μs, respectively. **D** Typical traces showing the delay time of APs change according to the intensity of local stimulus [keep stimulus amplitude constant (~35 V), change the duration]. First short-duration stimulus (50 μs, black), then long-duration stimulus (100 μs, red). **E** Quantification of (**D**). 14 cells for 50 μs and 100 μs, respectively. All recordings in each panel were performed in the same one cell and aligned based on the onset of the stimulus. The time interval between different stimulus is at least one min. Data are shown as mean + s.e.m; paired Student’s t test for panel C and E; **p < 0.01; ns, not significant

To validate whether above phenomenon generally exists in other nervous system, we repeated the same stimulating and recording protocol in hippocampal neurons (Fig. 3A). Consistently, the onset point of weak stimulus (with shorter duration) evoked PSC signal is more away from the stimulus artifact while strong stimulus (with longer duration) evoked PSC signal’s onset point is closer (Fig. 3B and C) in the same one neuron. Further, the presynaptic AP signals were analyzed and showed the same tendency (Fig. 3D and E), which suggests to explain the mechanism of varied onset points of PSC signals. Although the amplitude of PSC signal induced by strong stimulus is larger than that induced by weak stimulus, the amplitude of APs induced between weak and strong stimulus shows no significant change, which leaves the same question as in sensory system, that is, which factor determines the amplitude of synaptic transmission. Overall, apart from the sensory system, we further proved that stronger stimulation leads to greater secretion in a faster way in the hippocampus, which is suggested to be regulated by earlier started APs, implying broad applicability in the nervous system.

## Discussion

The precise coupling of neurotransmission and external stimulus is important for animals with nervous system to make effective response when faced with danger. Although the amplitude of neurotransmission is positively related to the intensity of external stimulus, whether stronger stimulus could trigger synaptic transmission faster remains unsolved. Our findings revealed that the intensity of stimulus determines the occurrence time of action potential (AP) and the following synaptic transmission. In other words, stronger stimulation induces earlier started AP, which suggests to trigger greater secretion in a faster way.

The major finding of this study is the discovery that stronger stimulus triggers synaptic transmission faster, which is supported by the following evidence: (i) Under stronger square-wave impulse with larger amplitude or (ii) longer duration, the recorded PSC signals’ onset point is much closer to the stimulus artifact (Fig. 1D-F) in the sensory system, indicating a faster occurrence of neurotransmission. (iii) What is more, our finding also works in the hippocampal system (Fig. 3B-C), implying broad applicability in the nervous system. Overall, our study showed that stronger stimulus triggers the neurotransmission faster.

The second major finding is that stronger stimulus induces earlier started presynaptic AP. The synaptic transmission is often correlated with APs [13, 21], which function to trigger the vesicle release and regulate the latency of synaptic transmission. To reveal whether AP regulates the different occurrence time of neurotransmission under different intensity of external stimuli here, we recorded and compared the presynaptic APs triggered by different stimuli. Our finding showed that stronger stimulus induces the AP more quickly in both the sensory system (Fig. 2B-C) and hippocampal system (Fig. 3D-E), which is much similar as the occurrence of neurotransmission, suggesting to explain the faster neurotransmission under stronger stimulus. To demonstrate the coupling between presynaptic AP and postsynaptic current, simultaneous paired recording [22] from synaptically pre- and postsynaptic compartments is needed in the future. In addition, the molecular mechanism of how the stimuli with different intensity regulate the occurrence of AP differentially is a fundamental question waiting to be revealed.

**Fig. 4:**
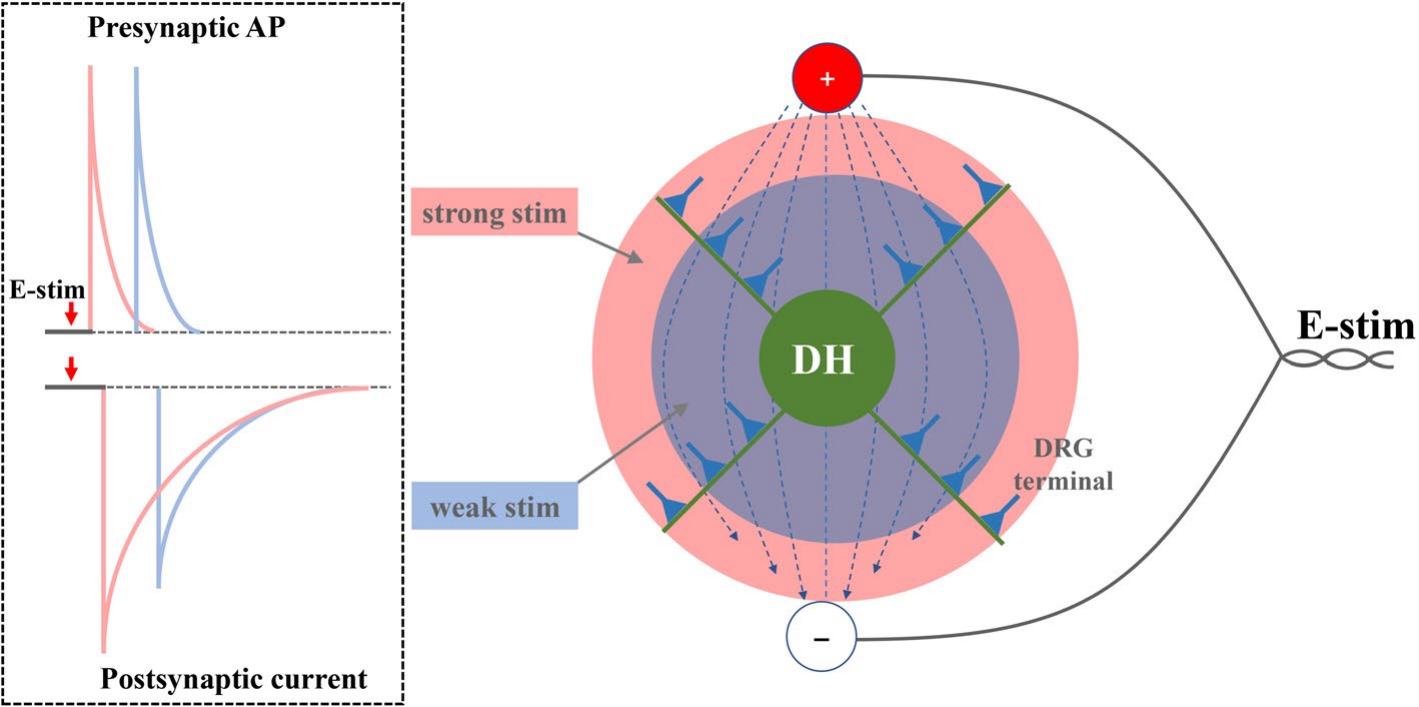
Model showing the regulation between the intensity of stimulus versus the occurrence time and amplitude of synaptic transmission. For the target postsynaptic DH neuron, it is within the electrical stimulus field (E-stim). The stronger stimulus can radiate bigger area and invade more presynaptic DRG terminals, which leads to larger synaptic transmission (showed as postsynaptic current, PSC). For individual presynaptic DRG terminal, the occurrence time of AP is determined by the intensity of received stimulus while its amplitude is constant. Stronger stimulation evoked AP faster in more DRG terminals, which suggests to trigger larger synaptic transmission in a faster way

Except for the occurrence time, the amplitude of the APs induced by different intensity of stimulus showed no significant change, which failed to explain the larger amplitude of neurotransmission under stronger stimulus. We then hypothesized that, for every individual synapse, the evoked AP’s amplitude may remain constant (in 0 or 1 digital form), which implies that the amplitude of single synaptic transmission triggered by single AP is constant. Under stronger stimulus, more synapses are invaded and the AP in individual synapse starts more quickly. We know that an action potential is generated when a stimulus changes the membrane potential to the values of threshold potential, which is usually around − 50 to − 55 mV. Then the threshold potential opens voltage-gated sodium channels and causes a large influx of sodium ions, which phase is called the depolarization. For the stimuli with different duration, actually the duration is equal to the stimulus strength. The stronger stimulus can change the membrane potential of more synapses to the threshold potential faster, which then induces the AP faster in more synapses, providing a plausible explanation for the larger and faster postsynaptic current signal (Fig. 4). However, our above hypothesis is far from perfect, which needs further experiments to demonstrate it. We then designed a plausible experiment to combine hippocampal neurons culture, the secretion reporter of synaptophysin-pHluorin [13] and confocal imaging with large field of view, with the predicted results that: stronger single pulse stimulus triggers more synaptic buttons’ fluorescence signal faster than weaker stimulus, while the ΔF/F_0_ fluorescence signal from each button shows no significant change.

In summary, based on the AP and PSC evidence from the primary sensory system and hippocampal system, we found that stronger stimulus triggered AP more rapidly, which suggested to induce synaptic transmission faster. Considering the importance of neurotransmission, our work suggests that the occurrence time of neurotransmission, as well as the amplitude, plays an important role in the nervous system to make corresponding reactions timely and effectively.

## Data Availability

All data generated or analyzed during this study are available upon request.

## Abbreviations

PSC: Postsynaptic current
AP: Action potential
DRG: Dorsal root ganglion neuron
DH: Spinal dorsal horn neuron
